# Elranatamab monotherapy in the real-word setting in relapsed-refractory multiple myeloma: results of the French compassionate use program on behalf of the IFM

**DOI:** 10.1038/s41408-024-01200-w

**Published:** 2024-12-18

**Authors:** Florent Malard, Arthur Bobin, Myriam Labopin, Lionel Karlin, Laurent Frenzel, Murielle Roussel, Marguerite Vignon, Sophie Godet, Thomas Chalopin, Perrine Moyer, Emilie Chalayer, Frederique Orsini Piocelle, Clara Mariette, Carolyne Croizier, Claudine Sohn, Mamoun Dib, Ronan Le Calloch, Nadia Ali-Ammar, Marion Loirat, Omar Benbrahim, Alexandre Payssot, Adrien Trebouet, Aurore Perrot, Xavier Leleu, Mohamad Mohty

**Affiliations:** 1 Sorbonne Université, Centre de Recherche Saint-Antoine INSERM UMRs938, Paris, France; 2 Service d’Hématologie Clinique et de Thérapie Cellulaire, Hôpital Saint Antoine, AP-HP, Paris, France; 3 Hematology, CIC 1082, U1313, CHU, University, Poitiers, France; 4 Centre Hospitalier Lyon Sud, Hospices Civils de Lyon, Pierre-Bénite, Lyon, France; 5 Hôpital Necker, Paris, France; 6 Centre Hospitalo-Universitaire Dupuytren, Limoges, France; 7 Hôpital, Cochin, Paris, France; 8 CHU de Reims, Reims, France; 9 CHU Tours, Tours, France; 10 CHU Nantes, Nantes, France; 11 Université Jean Monnet Saint-Étienne, CHU Saint-Étienne, INSERM, CIC1408, INSERM, SAINBIOSE-U1059, Service d’Hématologie Clinique et de Thérapie Cellulaire, F- 42023 Saint-Étienne, France; 12 CHR d’Annecy, Epagny Metz-Tessy, France; 13 CHU de Grenoble, La Tronche, France; 14 Service de Thérapie Cellulaire et d’Hématologie Clinique, CHU Estaing, EA 7453 CHELTER, Université Clermont Auvergne, Clermont-Ferrand, France; 15 Hôpital de Toulon, Toulon, France; 16 CHU d’Angers, Angers, France; 17 Cornouaille Hospital Center, Quimper, France; 18 Hôpital de Troyes, Troyes, France; 19 Hôpital Saint Nazaire, Saint-Nazaire, France; 20 CHU Orléans, Orléans, France; 21 Clinique du Parc, Castelnau Le Lez, France; 22 Bretagne Sud Hospital Centre, Lorient, France; 23 CHU de Toulouse, Toulouse, France

**Keywords:** Myeloma, Immunotherapy

Elranatamab is a humanized, bispecific antibody that targets B-cell maturation antigen (BCMA) on multiple myeloma (MM) cells and CD3 on T cells, with the aim of inducing T cell-mediated cytolysis of the MM cells [1, 2]. Elranatamab was approved as monotherapy for relapsed-refractory MM (RRMM) based on the phase II MagnetisMM-3 (NCT04649359) registrational study [3]. Here, we report clinical outcomes with standard-of-care elranatamab in a real-world RRMM population as part of the French compassionate use program.

A total of 101 patients from 22 centers who received elranatamab between 2022 and 2023 were included in this retrospective analysis, most of whom would have been considered ineligible for the registration trial (Table 1). Patients received step-up doses of 12 and 32 mg elranatamab subcutaneously on days 1 and 4 of cycle 1, respectively, followed by 76 mg elranatamab once-weekly, starting on day 8 of the first 4-week cycle. Treatment with elranatamab continued until disease progression, unacceptable toxicity, or withdrawal of consent.

**Table 1:** Patients’ characteristics and outcomes.

Characteristic	Study Population (*N* = 101)
Patient age, years, median (min-max) [IQR]	68 (39–87) [62–75]
Gender	
Male	52 (51%)
Female	49 (49%)
ECOG-PS	
0	16 (18%)
1	41 (47%)
≥ 2	30 (35%)
Missing	14
R-ISS at diagnosis	
1	24 (30%)
2	29 (36.2%)
3	27 (33.8%)
Missing	21
del(17p)	
No	46 (71%)
Yes	19 (29%)
Missing	36
t(4;14)	
No	45 (67%)
Yes	22 (33%)
Missing	34
Extra-medullary disease at elranatamab initiation	
No	57 (65%)
Yes	30 (36%)
Missing	14
Number of previous lines, median (min-max) [IQR]	5 (1–17) [3–6]
Triple-class exposed (at least 1 PI, 1 IMiD, 1 anti-CD38 mAb)	
No	4 (4%)
Yes	97 (96%)
Penta-exposed (at least 2 PI, 2 IMiD, 1 anti-CD38 mAb)	
No	24 (24%)
Yes	77 (76%)
Triple-class refractory (at least 1 PI, 1 IMiD, 1 anti-CD38 mAb)	
No	48 (48%)
Yes	53 (53%)
Penta-refractory (at least 2 PI, 2 IMiD, 1 anti-CD38 mAb)	
No	79 (78%)
Yes	22 (22%)
Previous anti-BCMA	
No	84 (83%)
Yes	17 (17%)
Time from prior treatment line end date to elranatamab, days, median (min-max) [IQR]	41 (2–2727) [27–71]
Median follow-up, months, median (min-max)	15.5 (3.4–18.8)
Overall reponse rate	52 (52%)
Best response	
Minimal response	2 (2%)
Partial response	10 (10%)
Very good partial response	23 (23%)
Complete response	16 (16%)
Stringent complete response	3 (3%)
≥ VGPR after one cycle	22 (22%)
At one year	
Overall survival	42% (95% CI: 31.7–52.1)
Progression-free survival	34% (95% CI: 24.4–43.7)
Duration of response	48% (95% CI: 31.1–64.0)
Time to next treatment	34% (95% CI: 24.2–43.6)
Progression following elranatamab therapy	
No	53 (53%)
Yes	48 (47%)
Subsequent lines of treatment (after 1st progression or elranatamab discontinuation)	
No	71 (74%)
Yes	25 (26%)
Missing	5

*ECOG-PS* Eastern Cooperative Oncology Group-Performance Status, *R-ISS* revised International Staging System, *min-max* minimum-maximum, *IQR* Interquartile range, *PI* proteasome inhibitor, *IMiD* immunomodulatory drug, *anti-CD38* mAb monoclonal antibody, *BCMA* B-cell maturation antigen, *VGPR* very good partial response.

Median age was 68 (range, 39–87) years. Median time from myeloma diagnosis to elranatamab administration was 75 (range, 16–239) months. Eighty percent had a revised International Staging System (R-ISS) of II or III, and 29% harbored del(17p), while 35% had extra-medullary disease. Of note, 8% had a creatinine clearance < 30 mL/min. Thirty-five percent of patients had an Eastern Cooperative Oncology Group-Performance Status (ECOG-PS) ≥ 2. Patients received a median of 5 (range, 1–17) prior lines of therapy, and the median time from date of prior line of treatment discontinuation to elranatamab was 41 days. Ninety-six percent were triple-class exposed, 76% were penta-exposed, and 17% had received prior BCMA-directed therapy.

In this real-world study, the overall response rate (ORR) was 51.5% (*n* = 52), and the complete response (CR) or very good partial response (VGPR) rate was 42% (*n* = 42). Response to elranatamab was quick with 22% of patients achieving ≥VGPR after only one cycle. With a median follow-up of 15.5 (range, 3.4–18.8) months, the one-year progression-free survival (PFS) and overall survival (OS) rates were 34% (95% confidence interval [CI]: 24.4–43.7) and 42% (95% CI: 31.7–52.1), respectively, while the one-year duration of response was 48% (95% CI: 31.1–64) (Fig. 1A). At last follow-up, 48 (47.5%) patients progressed following elranatamab therapy and 25 (25%) patients managed to receive a subsequent line of therapy after progression or elranatamab discontinuation. The one-year time to next treatment was 34% (95% CI: 24.2–43.6) (Fig. 1B). In univariate analysis, ECOG-PS ≥ 2 and male patient sex were associated with a lower ORR. The ORR was 87.5%, 53.7%, and 30% for ECOG-PS = 0, 1, and ≥ 2, respectively (*p* = 0.0008). For male versus female, the ORR was 40.4% versus 63.3%, respectively (*p* = 0.021). Importantly, previous anti-BCMA exposure and extra-medullary disease were not associated with a significantly lower ORR (Supplementary Table 1). In multivariate logistic regression analysis, the only parameter associated with patients’ outcomes was an ECOG-PS ≥ 2, which was predictive of a lower ORR (hazard ratio [HR] = 0.065, 95% CI: 0.009–0.30; *p* = 0.0018) and a decreased PFS (HR = 3.06, 95% CI: 1.31–7.15; *p* = 0.01) and OS (HR = 8.32, 95% CI: 2.49–27.81; *p* = 0.0006) (Supplementary Table 2).

**Fig. 1:**
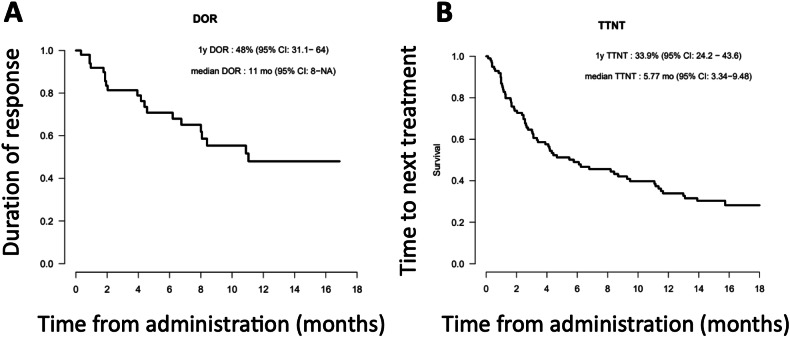
Real-world clinical outcomes with elranatamab. **A** Duration of response; (**B**) Time to next treatment.

Regarding safety outcomes, cytokine release syndrome (CRS) was observed in 45% of patients, and no event was grade ≥ 3, whereas immune effector cell-associated neurotoxicity syndrome (ICANS) was observed in only 3 (3%) of patients (all were grade 1-2) (Supplementary Table 3). Forty-nine percent of patients experienced at least 1 infection, with 24 patients (24%) having infections graded as severe (grade ≥ 3). Forty-four viral infections were reported, including 20 of which were grade ≥ 3 (Supplementary Table 4). Grade 1-2 aspergillosis was reported in 6 patients (6%) and grade 1 oropharyngeal candidiasis in 1 patient (1%). Of note, no toxoplasmosis due to *Pneumocystis jiroveccii* infection was reported. Finally, 19 bacterial infections (grade ≥ 3, *n* = 8) and 11 undocumented infections were reported (grade ≥ 3, *n* = 4) (results not shown). Only 50% of patients received intravenous immunoglobulin supplementation during elranatamab therapy.

In this compassionate program analysis, we report an ORR to elranatamab of 51.5% which appears to be lower compared to the ORR of 61.0% reported in the MagnetisMM-3 study [3]. Of note, 17% of patients received prior anti-BCMA-directed therapy in our cohort, with an ORR of 47.1%, which may have contributed marginally to the lower ORR observed in our study and was comparable to the ORR of 53.8% reported in the MagnetisMM-1 phase 1 study, in patients with prior BCMA-directed therapy [1]. Furthermore, while cohorts were nearly equal regarding extra-medullary disease and cytogenetic status, 35% of patients in our cohort had an ECOG-PS ≥ 2, versus only 6% of patients with an ECOG-PS 2 in the MagnetisMM-3 study. Importantly, we found that, apart male sex, an ECOG-PS ≥ 2 was the only parameter associated with a lower ORR in multivariate analysis, making this high proportion of patients with an ECOG-PS ≥ 2 the leading cause of the lower ORR in our cohort compared to the prospective MagnetisMM-3 study. Importantly, ECOG-PS ≥ 2 was also associated in multivariate analyses with lower OS and PFS, as reported in previous studies [4, 5]. Our findings were also comparable to the real-world data on teclistamab in RRMM with an ORR of 59.3 and 62%, in Germany and the United States (US), respectively [6, 7]. Of note, ECOG-PS status was not available for this real-world data on teclistamab.

Regarding toxicity, we observed 45% of grade 1–2 CRS with no severe CRS, and 3% of ICANS, which was comparable to the findings of the prospective MagnetisMM-3 study with an incidence of CRS and ICANS of 56.3% and 3.4%, respectively [3], despite a significant number of frail patients with an ECOG-PS ≥ 2 in our cohort. Furthermore, given the high incidence of infectious complications reported with bispecific antibodies that target BCMA [8], we rigorously evaluated their incidence in our cohort. All grade, and grade ≥ 3 infections were reported in 49 and 24% of the patients, respectively, which compared favorably with the 69.9% and 39.8%, respectively, reported in the MagnetisMM-3 study, and was comparable to the findings in the real-world cohorts on teclistamab (German cohort 54.5% and 26.8%, respectively [6]; United States cohort 40 and 26%, respectively [7]).

Overall, our cohort including patients with highly advanced disease, a significant proportion of whom had received prior anti-BCMA directed therapy, extra-medullary disease, and adverse prognostic features, particularly poor ECOG-PS, confirms that elranatamab was safe and effective in RRMM.

## Supplementary information

SUPPLEMENTAL MATERIAL
