# Supplementary material for: Elranatamab monotherapy in the real-word setting in relapsed-refractory multiple myeloma: results of the French compassionate use program on behalf of the IFM
Source: Blood Cancer J. 2024 Dec 18;14(1):219. doi: 10.1038/s41408-024-01200-w (PMC11655550; doi:10.1038/s41408-024-01200-w)
Supplement: Supplementary file 1 — SUPPLEMENTAL MATERIAL [file 41408_2024_1200_MOESM1_ESM.docx]

**Elranatamab monotherapy in the real-word setting in relapsed-refractory multiple myeloma: results of the French compassionate use program on behalf of the IFM**

Florent Malard^1^, Arthur Bobin^2^, Myriam Labopin^1^, Lionel Karlin^3^, Laurent Frenzel^4^, Murielle Roussel^5^, Marguerite Vignon^6^, Sophie Godet^7^, Thomas Chalopin^8^, Perrine Moyer^9^, Emilie Chalayer^10^, Frederique Orsini Piocelle^11^, Clara Mariette^12^, Carolyne Croizier^13^, Claudine Sohn^14^, Mamoun Dib^15^, Ronan Le Calloch^16^, Nadia Ali-Ammar^17^, Marion Loirat^18^, Omar Benbrahim^19^, Alexandre Payssot^20^, Adrien Trebouet^21^, Aurore Perrot^22^, Xavier Leleu^2^, Mohamad Mohty^1^

1. Sorbonne Université, Centre de Recherche Saint-Antoine INSERM UMRs938, Paris, France ; Service d'Hématologie Clinique et de Thérapie Cellulaire, Hôpital Saint Antoine, AP-HP, Paris
2. Hematology, CIC 1082, U1313, CHU, University, Poitiers,
3. Centre Hospitalier Lyon Sud, Hospices Civils de Lyon, Pierre-Bénite
4. Hôpital Necker, Paris
5. Centre Hospitalo-Universitaire Dupuytren, Limoges
6. Hôpital, Cochin, Paris
7. CHU de Reims
8. CHU Tours
9. CHU Nantes
10. Université Jean Monnet Saint-Étienne, CHU Saint-Étienne, INSERM, CIC1408, INSERM, SAINBIOSE-U1059, Service d'Hématologie Clinique et de Thérapie Cellulaire, F- 42023 Saint-Étienne
11. CHR d'Annecy, METZ TESSY
12. CHU de Grenoble
13. Service de Thérapie Cellulaire et d’Hématologie Clinique, CHU Estaing, EA 7453 CHELTER, Université Clermont Auvergne, Clermont-Ferrand
14. Hôpital de Toulon
15. CHU d’Angers
16. Cornouaille Hospital Center, Quimper
17. Hôpital de Troyes
18. Hôpital Saint Nazaire
19. CHU Orléans
20. Clinique du Parc, Castelnau Le Lez
21. Bretagne Sud Hospital Centre, Lorient
22. CHU de Toulouse, France.

**Supplementary file**

**Supplementary Table 1. Univariate analysis of overall response rate.**

|  |  | ORR | |
| --- | --- | --- | --- |
|  |  | % | p value |
| Patient sex | Male | 40.4% |  |
|  | Female | 63.3% | 0.021 |
| ECOG-PS | 0 | 87.5% |  |
|  | 1 | 53.7% |  |
|  | 2 to 5 | 30% | 0.0008 |
| Patient age | <75 years | 50.7% |  |
|  | >=75 years | 53.8% | 0.78 |
| Baseline creatinine clearance (ml/min) | <69 (median) | 51.0% |  |
|  | >69 | 51.0% | 1 |
| Extra-medullary disease | No | 52.6% |  |
|  | Yes | 43.3% | 0.41 |
| Prior BCMA exposure | No | 52.4% |  |
|  | Yes | 47.1% | 0.69 |

Abbreviations: ORR, overall response rate; ECOG-PS, Eastern Cooperative Oncology Group-Performance Status; BCMA, B-cell maturation antigen.

**Supplementary Table 2. Multivariate analysis of overall response rate, progression-free survival and overall survival.**

|  |  | ORR | | PFS | | OS | |
| --- | --- | --- | --- | --- | --- | --- | --- |
|  |  | HR (95% CI) | p value | HR (95% CI) | p value | HR (95% CI) | p value |
| Patient sex | Male (reference) | 1 |  | 1 |  |  |  |
|  | Female | 3.10 (1.19-8.51) | 0.023 | 0.73 (0.43-1.24) | 0.25 | 0.77 (0.44-1.32) | 0.34 |
| ECOG-PS | 0 (reference) | 1 |  | 1 |  | 1 |  |
|  | 1 | 0.174 (0.02-0.76) | 0.037 | 1.51 (0.65-3.54) | 0.34 | 3.57 (1.07-11.98) | 0.039 |
|  | 2 to 5 | 0.065 (0.009-0.30) | 0.0018 | 3.06 (1.31-7.15) | 0.01 | 8.32 (2.49-27.81) | 0.0006 |
| LP score>1 or del(17p) or t(4;14) | Absent or unknown (reference) | 1 |  | 1 |  |  |  |
|  | Present | 0.70 (0.26-1.90) | 0.49 | 1.52 (0.89-2.59) | 0.12 | 1.56 (0.89-2.74) | 0.12 |
| Patient age | <75 years (reference) | 1 |  | 1 |  |  |  |
|  | >=75 years | 1.47 (0.48-4.70) | 0.50 | 1.3 (0.71-2.39) | 0.4 | 1.55 (0.82-2.94) | 0.18 |

Abbreviations: ORR, overall response rate; PFS, progression-free survival; OS overall survival; HR, hazard ratio; ECOG-PS, Eastern Cooperative Oncology Group-Performance Status; linear predictor.

**Supplementary Table 3. Side effects.**

| Side effect | Study Population (N=101) |
| --- | --- |
| Cytokine release syndrome  Grade 1  Grade 2  Grade3  Grade 4  Grade 5 | 35 (35%)  10 (10%)  0  0 |
| Immune effector cell-associated neurotoxicity syndrome  Grade 1  Grade 2  Grade3  Grade 4  Grade 5 | 1 (1%)  2 (2%)  0  0  0 |
| Infections  Severe infection (grade ≥ 3) | 50 (49%)  24 (24%) |
| Intravenous immunoglobulin supplementation | 51 (50%) |

**Supplementary Table 4. Viral infections**

| Infectious complication | Study Population (N=101) |
| --- | --- |
| COVID-19  All grade  Grade ≥3 | 15  7 |
| Cytomegalovirus reactivation  All grade  Grade ≥3 | 11  7 |
| Rhinovirus  All grade  Grade ≥3 | 5 (%)  2 |
| Influenza  All grade  Grade ≥3 | 2  1 |
| Respiratory syncytial virus  All grade  Grade ≥3 | 2  1 |
| Metapneumovirus  All grade  Grade ≥3 | 2  1 |
| Enterovirus  All grade  Grade ≥3 | 2  0 |
| Parvovirus  All grade  Grade ≥3 | 2  1 |
| Parainfluenza  All grade  Grade ≥3 | 1  0 |
| Hepatitis B virus reactivation  All grade  Grade ≥3 | 1  0 |
| Human herpes virus type 6 reactivation  All grade  Grade ≥3 | 1  0 |
